# A Portable Tunable Diode Laser Absorption Spectroscopy System for Dissolved CO_2_ Detection Using a High-Efficiency Headspace Equilibrator

**DOI:** 10.3390/s21051723

**Published:** 2021-03-02

**Authors:** Zhihao Zhang, Meng Li, Jinjia Guo, Baolu Du, Ronger Zheng

**Affiliations:** College of Information Science and Engineering, Ocean University of China, Qingdao 266100, China; zhihaozhang@stu.ouc.edu.cn (Z.Z.); limeng@stu.ouc.edu.cn (M.L.); dbl@stu.ouc.edu.cn (B.D.); rzheng@ouc.edu.cn (R.Z.)

**Keywords:** dissolved CO_2_, TDLAS, underway measurement, headspace equilibrator, portable system

## Abstract

Continuous observation of aquatic $pCO_{2}\;$ at the ocean surface, with a sensitive response time and high spatiotemporal resolution, is essential for research into the carbon biogeochemical cycle. In this work, a portable tunable diode laser absorption spectroscopy (TDLAS) system for dissolved CO_2_ detection in surface seawater, coupled with a home-made headspace equilibrator, allowing real time underway measurements, is described. Both the optical detection part and sample extraction part were integrated together into a compact chamber. An empirical equation suitable for this system was acquired, which can convert the concentration from the gas-phase to the aqueous-phase. A monitoring precision of 0.5% was obtained with time-series measurement, and the detection limits of 2.3 ppmv and 0.1 ppmv were determined with 1 s and 128 s averaging time, respectively. Sampling device used in this work was ameliorated so that the response time of system reduced by about 50% compared to the traditional ‘shower head’ system. The fast response time reached the order of 41 s when the final concentration span was 3079 ppmv. For1902 ppmv, this figure was as short as 20 s. Finally, a field underway measurement campaign was carried out and the results were briefly analyzed. Our work proved the feasibility of the TDLAS system for dissolved CO_2_ rapid detection.

## 1. Introduction

The present-day (2020) atmospheric carbon dioxide (CO_2_) levels of more than 410 ppmv are nearly 50% higher than preindustrial concentrations, and the current elevated levels and rapid growth rates are unprecedented in the past 55 million years of the geological record [1,2]. The oceans, which account for 71% of the Earth’s surface area, represent a significant sink of anthropogenic CO_2_ emissions, making remarkable sense to global carbon cycle and in mitigating anthropogenic climatic anomalies [3,4,5]. Some 30% of total anthropogenic CO_2_ emissions were absorbed by the oceans [4,6] and the rate of ocean uptake of atmospheric CO_2_ has increased continuously for the last two decades on account of the ever-increasing concentration of CO_2_ in the atmosphere [7]. The anthropogenic carbon dioxide emissions have caused pronounced changes to the marine carbonate system [8], and are altering the surface seawater acid-base chemistry towards increased acidic globally [1,9]. However, many potential biogeochemical feedbacks from increasing seawater CO_2_ are not well understood entirely, with quantities of high-quality data required to improve the understanding of internal mechanisms [8,9]. In order to understand the ocean’s role in the uptake of atmospheric CO_2_ and thus the effects on climate and ocean carbon cycling, as a primary approach, the air-sea exchange of CO_2_ in the surface seawater must be measured [10]. The partial pressure of CO_2_ ($pCO_{2}$) at the ocean surface varies geographically and seasonally over a wide range [11]. Especially in some regions where $pCO_{2}$ varies strongly, data with a higher resolution are needed to resolve the spatiotemporal variability of $pCO_{2}\;$ fluxes [12]. Therefore, the continuous underway measurement of the spatial distribution and temporal variability of $pCO_{2}$ dissolved in seawater, with high data point density, is essential and beneficial for understanding the ocean carbon biogeochemical cycle.

To understand the complex processes of the carbon cycle at the sea-air interface, a crucial step is to implement real-time observation of dissolved $pCO_{2}$ in surface seawater. Several geochemical analytical approaches have been successfully employed for this purpose [13].

As a mature method, gas chromatography (GC)-based $pCO_{2}$ detection has proven itself via years of successful deployments in aqueous system because its low air sample consumption, a linear response over a wide range of concentrations, and high precision [14,15,16]. However, the application of GC-based $pCO_{2}$ detection system in aqueous environment is limited by its poor sampling density [17], caused by time-consuming discrete sampling, strict requirements for stable temperatures, expensive catalysts for analysis, sample pollution during sampling, and time delays [8,18,19]. In marine research, membrane injection mass spectrometry (MIMS)-based $pCO_{2}$ detection is also a typical approach used in aqueous environment observation tasks, such as monitoring of air-sea $pCO_{2}$ fluxes [20] and oceanic inorganic carbon [21], allowing precise real time monitoring. Nevertheless, application of MIMS in underway measurements is limited by the enormous bulk of the instrumentation, high cost, complex sample preprocess operations and the membrane injection technique used [14].

In comparison to the approaches mentioned above, optical methods have many advantages in gas detection, and have gradually become a research hotspot. Non-dispersive infrared (NDIR)-based technology is increasingly being deployed in observation of $pCO_{2}$ [22,23], yet, its applications are limited by the poor selectivity caused by the use of a wideband light source. As a technology that can realize in-situ measurements, a probe based on Raman spectroscopy has been successfully deployed for the detection of bicarbonate in hydrothermal areas [24], but for the special case of dissolved CO_2_ detection, its sensitivity is insufficient. In addition, cavity ring-down spectroscopy (CRDS)-based technology and off-axis integrated cavity output spectroscopy (OA-ICOS)-based technology are also increasingly being recognized and used in continuous CO_2_ and CH_4_ measurements both in surface water and atmosphere [25,26]. Despite the high sensitivity of sensors based on OA-ICOS and CRDS, however, they require rather complex setups with laser frequency locking to longitudinal cavity resonances [27].

Tunable diode laser absorption spectroscopy (TDLAS), based on molecule infrared spectroscopy, can distinguish diverse molecules precisely because absorption lines of the target gas are selected characteristically. It is advantageous for trace gas sensing in terms of cost performance, stability, environmental adaptability and no pretreatment. For the case of CO_2_, the background concentration in the atmosphere and seawater can reach the order of several hundreds of ppmv, which meant that the detection sensitivity of TDLAS is adequate for CO_2_ measurement, so TDLAS-based systems have a better cost performance than systems based on CRDS or ICOS. With remarkable response time and sensitivity, numerous employments have demonstrated that TDLAS is a potential alternative tool for underway measurement of $pCO_{2}$ in surface seawater [13,28]. Combined with an appropriate gas-liquid separation device, the ability to measure at a high temporal and spatial resolution is obtained, allowing the dynamic variation of aquatic $pCO_{2}$ to be revealed and regions with significant changes in carbonate system to be identified [8]. Despite these many superior features, however, TDLAS-based underway measurement of oceanic $pCO_{2}$ has rarely been reported.

With the aim of developing an underway measurement system which can capture the spatial-temporal distribution and dynamic variation of dissolved CO_2_ in ocean surface waters, a deck-based TDLAS system combined with a specially designed gas extraction device was developed. Both the optical detection part and sample extraction part were integrated into a compact chamber together that can realize dissolved CO_2_ measurements directly. A multi-path gas cell (MPGC) was designed to achieve an optical path length of more than 11 m and an internal volume of less than 80 mL considering the need of small volume and long optical path length. In the special case of underway measurements, the headspace equilibrator was designed to work simultaneously with two methods of spray and bubble so that the response time of system was reduced by about 50% compared to traditional “shower head” headspace equilibrator. A series of experiments carried out in the laboratory were conducted to verify the reliability and performance of the system for aquatic CO_2_ detection. Finally, a field application of continuous underway monitoring of oceanic $pCO_{2}$ was deployed in Jiaozhou Bay, Qingdao, China. The results and details are discussed in this paper. Our work proved the feasibility of the TDLAS-based system for dissolved CO_2_ rapid detection, and can provide a reference for other researchers engaged in marine sensor development.

## 2. Apparatus and Methods

The developed dissolved CO_2_ detection system consisted of two main functional parts: the optical detection part and the sample extraction part. Both the above parts were fitted into a portable aluminum alloy chamber which was 40 cm in height, 50 cm in length and 30 cm in width. In this way, the gas pipeline volume was reduced and the response time was improved. In addition, this design could avoid the influence of high humidity and salinity on the optical and circuit devices. During underway measurements, the system can be powered through marine electric equipment, allowing long-term deployment.

**Figure 1:**
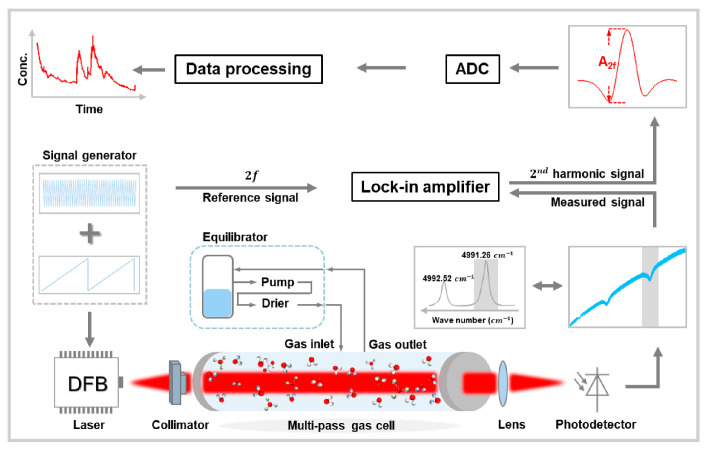
Schematic diagram of the optical detection part of the developed dissolved CO_2_ detection system. DFB: distributed feedback laser.

The schematic diagram of the optical design is depicted in Figure 1. In this work, the absorption line at 4991.26 cm^−1^ was selected [29,30], which had a proper absorption intensity and no other interferential gases nearby. The optical detection part was based on the mature and traditional technology of TDLAS, using a continuous wave tunable diode laser (DFB-2004-3, nanoplus GmbH, Gerbrunn, Free State of Bavaria, Germany) operating at room temperature as the light source. Such a tunable laser output light with a single frequency and a narrow linewidth at the absorption line of target gas molecule, which was selected characteristically. In this way, TDLAS can distinguish diverse molecules precisely and provide an excellent selectivity for the detected gases. Under the tuning of current and temperature, the central wavelength of the laser scanned over a small range with a certain frequency (50 Hz in this work), so that the characteristic absorption line of the target gas was covered. The laser current was set at 80 mA with a temperature set at 3 °C for targeting the selected CO_2_ absorption line. Along the laser beam path, laser spectrum was modified by the light absorption at the wavelength of selected absorption line, and the concentration of target gas was obtained after the laser beam captured by the detector. The fundamental principle of TDLAS, Beer–Lambert law, indicated that the detection sensitivity was proportional to effective optical path length and molecular absorption line intensity, so that a MPGC was usually used to acquire a higher sensitivity. Both the increase of gas concentration and optical path in the MPGC can increase the intensity of particulate scattering (Rayleigh scattering). However, different from TDLAS, the Rayleigh scattering did not have wavelength selectivity, which just had the influence on the background of TDLAS second harmonic signal. And the intensity of Rayleigh scattering was small because it was inversely proportional to the fourth power of the wavelength. Therefore, the influence of particle scattering was ignored in this work. Additionally, tunable diode laser absorption spectroscopy-wavelength modulation spectroscopy (TDLAS-WMS), used extensively for weak signal detection, was another effective method to improve the sensitivity.

The diode current and temperature of the laser were controlled by a commercial TEC controller (CLD1015, Thorlabs Inc., Newton, NJ, USA). With the injection of a mixed current of low-frequency sawtooth wave (50 Hz) and high-frequency sine wave (15 kHz), the laser wavelength was modulated. In order to meet the demands of miniaturization and high precision, a customized MPGC designed with an improved Herriott type was used in this work. The MPGC consisted of two 2″ silver coated concave mirrors (d = 1 inch, f = 50 mm, R > 97.5% at 2004 nm) separated by a distance of 10.3 cm, which allows the laser beam to pass 113 times between two mirrors (leading to an effective optical pathlength of ~11.6 m). Two mirrors, aligned parallel to each other, were enclosed within the MPGC of 3.2 cm inner diameter and 11 cm height, resulting in a sample volume of less than 78 mL at 1 atm (the volume of the internal structural metal was removed). Such a small volume inside the MPGC could ensure a fast-dynamic response when measurement occurs under a certain pressure and gas velocity. To realize system integration, the gas inlet, gas outlet, pressure sensor, and temperature sensor were integrated on the sidewall of the MPGC. To achieve air impermeability, two rubber O-rings were used to connect the cavity, flange and top cover. On the top cover, we used BaF_2_ as the optical window material, with a transmittance of 94% at 2004 nm, thus ensuring both tightness and transmitted light intensity. In order to eliminate the negative effect on the system accuracy, optical path outside the MPGC was designed to be sealed and filled with pure nitrogen, so as to keep the CO_2_ concentration in this part was relatively constant. 

The laser beam collimated with a fiber-coupled collimator subsequently injected the MPGC at a certain angle and position. Then, a fixed reflection mode formed inside the MPGC, thus increasing the absorption path and improving the detection sensitivity. The demodulation of the second harmonic signal (2f-signal) was performed by a lock-in amplifier (LIA-MV-200-H, FEMTO Messtechnik GmbH, Berlin, Germany). A low power consumption, high performance embedded industrial computer (PCM-3365, Advantech Technology (China) Co., Ltd., Kunshan, Jiangsu, China) was used as the storage unit and operation carrier of the host system software.

**Figure 2:**
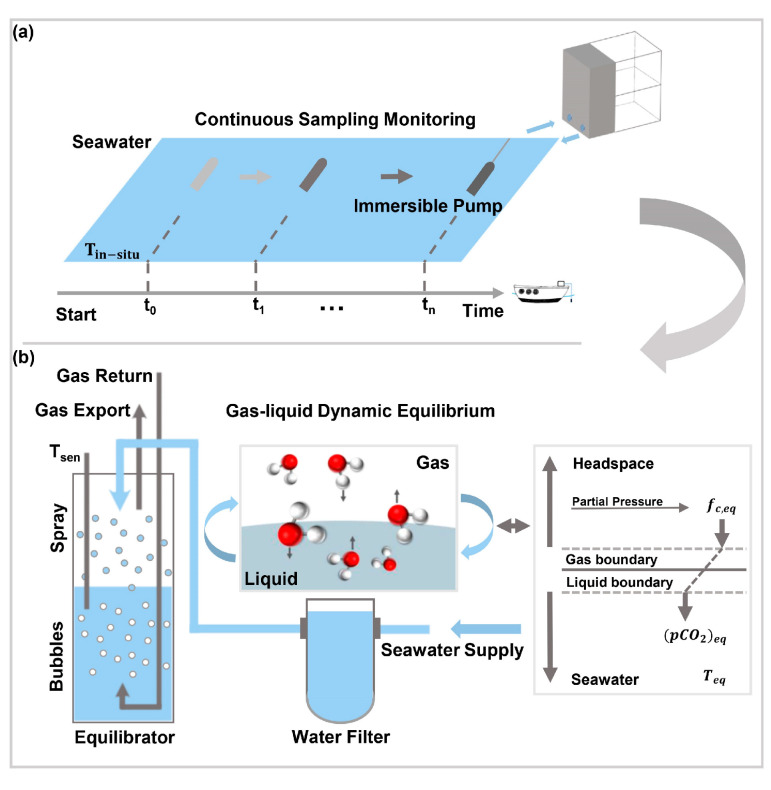
Schematic diagram of the sample extraction part of the system. (**a**) Continuous underway observation of the surface seawater. (**b**) Details of the headspace equilibrator and gas exchange model between the two gas-liquid phases. Gas Return: gas inlet for waste gas measured in the MPGC; Gas Export: gas outlet for equilibrium gas;$\;T_{sen}$: temperature sensor of the water sample in the equilibrator.

The ocean is a complicated aqueous system, and in many cases, sampling density is significant for marine underway measurements because low-frequency data cannot capture the dynamic variations of dissolved CO_2_. To obtain data with a high temporal-spatial resolution, a home-made sample extraction device based on a headspace equilibrator was established. It was able to realize continuous sampling in marine surface water, as shown in Figure 2a.

Different from the traditional “shower head” sampler which just sprays droplets in the top space for gas-liquid equilibrium, the sampling device deployed in this study was specially designed with two methods of gas-liquid equilibrium—spray and bubble—as shown in Figure 2b. Equilibrium gases were pumped from the “gas export” to the MPGC, where the concentration in the gas-phase could be acquired. The exhaust gas was reversed back to the “gas return” inlet, and was used to generates a large number of bubbles through the bubble generator, which are pumped from the MPGC to the bottom water of the equilibrator. Tiny droplets jetted from the top of the headspace and bubbles emerged from the bottom, effectively increasing the contact area between the gas and liquid. In this way, gas-liquid separation efficiency was improved and the response time of system was shortened. Thus, gases between the dual-phase could reach equilibrium more quickly than with a “shower head” device reported before [31]. $T_{sen}$ was the temperature sensor for the water sample in the equilibrator. The flow rate of the sample gas, which was related to the internal pressure of the MPGC, was controlled through a mass flowmeter before entering the MPGC. A vapor filter was employed to remove the influence of the vapor. 

As a classical method for sample extraction of dissolved gas, a headspace equilibrator is often used to study the air-sea exchange while an underway measurement is carried out [32]. The right side of Figure 2b, inset, shows the gas exchange model between the air and liquid [33]. For the special case in the equilibrator (left side of Figure 2b), gases dissolved in water and gases in the headspace exchange mutually until they reach dynamic equilibrium. Dissolved gases in the liquid-phase are striped into the gas-phase. If the difference between the in-situ temperature of seawater ($T_{in-situ},\;℃$) and the temperature of the equilibrator ($T_{eq},\;℃$) is less than 2 ℃, especially during the underway measurement, the $pCO_{2}$ at the in-situ temperature (${\left(pCO_{2}\right)}_{sw},\;\mu atm$) could be calculated by the formula from Takahashi et al. [18,34], as shown in Equation (1):

(1) $${\left(pCO_{2}\right)}_{sw}={\left(pCO_{2}\right)}_{eq}\times exp\left[0.0423\left(T_{in-situ}-T_{eq}\right)\right],\;$$

The $pCO_{2}$ at equilibration temperature (${\left(pCO_{2}\right)}_{eq},\mu \mathrm{atm}$) can be expressed in terms of partial pressure in the gas-phase ($f_{c,eq},\;ppmv$) while under equilibrium state using Equation (2) [35]:

(2) $${\left(pCO_{2}\right)}_{eq}=f_{c,eq}\times \left[{\left(P_{b}\right)}_{eq}-{\left(P_{H_{2}O}\right)}_{eq}\right],\;$$

where $f_{c,eq}$ was the $CO_{2}$ mole fraction in dry air that equilibrated with the water sample and barometric pressure (${\left(P_{b}\right)}_{eq}$) in the equilibrator after correcting for the vapor pressure (${\left(P_{H_{2}O}\right)}_{eq}$) at 100% relative humidity [36]. The $f_{c,eq}$ was the function of the amplitude of the 2f-signal ($A_{2f},\;f_{c,eq}\propto A_{2f}$). In addition, the value of the vapor partial pressure was calculated by Equation (3) at $T_{eq}$ and in-situ salinity (S‰) [35]. In this work, the direct measurement quantity we obtained was $A_{2f}$, which can be converted into $f_{c,eq}$ after the system is calibrated:

(3) $$\ln{\left(P_{H_{2}O}\right)}_{eq}=24.4543-67.4509\left(100/T_{eq}\right)-4.8489\ln\left(T_{eq}/100\right)-0.000544S‰$$

## 3. Results and Discussion

### 3.1. System Calibration

To achieve quantitative monitoring of dissolved CO_2_, one should pay particular attention to calibration of the device under controllable conditions in the laboratory. In this experiment, we used four dry and certified calibration gases, whose concentrations were respectively 398 ppmv, 808 ppmv, 1006 ppmv, and 2019 ppmv, to calibrate the system. These calibration gases consisted of carrier gases of nitrogen and carbon dioxide. In this experiment, we pumped the MPGC below 700 Torr and flushed it with pure nitrogen gas for 20 min. In this way, the background noise level was obtained. Then, we sequentially filled the chamber with the calibration gases mentioned above and obtained the amplitude of the 2f-signal ($A_{2f}$) averaged over 5 min. Obviously, the accuracy of the measurement results depends on good air-tightness and stable pressure within the MPGC. According to the data, the dependence of $A_{2f}$ and $f_{c,eq}$ was fitted as the following equation, with the R-squared value > 0.99:

(4) $$f_{c,eq}=3800.65\times A_{2f}-142.35,\;\mathrm{in}\;\mathrm{ppmv}$$

After the calibration model of this system was established in Equation (4), the $A_{2f}$ could be transformed into the gas-phase concentration ($f_{c,eq}$). For the TDLAS system, the empirical formula applicable to the calculation of ${\left(pCO_{2}\right)}_{sw}$ was obtained through the above formulas, as expressed in Equation (5). The ${\left(pCO_{2}\right)}_{sw}$ was the function of $A_{2f}$, ${\left(P_{b}\right)}_{eq}$, $T_{eq}$, $T_{in-situ}$ and S. Obviously, this method relied on the precise quantification of $A_{2f}$ [14]:

(5) $${\left(pCO_{2}\right)}_{sw}=\left(3800.65\times A_{2f}-142.35\right)\times \left\{{\left(P_{b}\right)}_{eq}-exp\left[24.4543-67.4509\left(100/T_{eq}\right)-\;4.8489\ln\left(T_{eq}/100\right)-0.000544S‰\right]\right\}\times exp\left[0.0423\left(T_{in-situ}-T_{eq}\right)\right],\;$$

**Figure 3:**
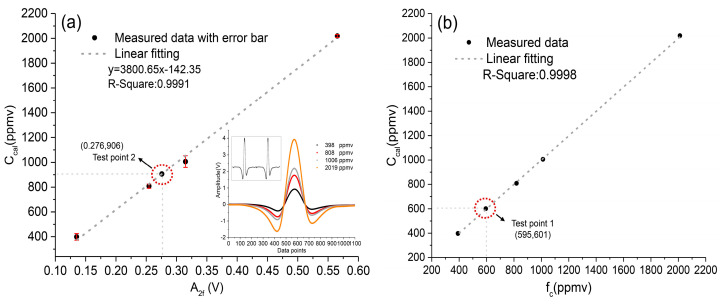
The measured 2f-signal versus corresponding concentrations of calibration gases. (**a**) System calibration experiment with four calibration gases. C_cal_: concentration of calibration gases. (**b**) Verification results of the system calibration model. fc: gas-phase concentration; Test point 1: another standard gas of 601 ppmv used as a checkpoint.

In order to verify the reliability of this model, the above calibration gases were measured again. In addition, we measured another standard gas of 601 ppmv as a checkpoint (as “Test point 1” in Figure 3b), and the measured value was 595 ppmv. The linear fitting result is shown in Figure 3b. There was a good agreement between $f_{c,eq}$ and the corresponding calibration gas concentration ($C_{cal}$), with a R-squared value more than 0.999. Within the range of 398–2019 ppmv, the fitting relative error of the CO_2_ concentration ((measured value-standard value)/standard value) was less than 2%, and the fluctuation range was from 1.7% (398 ppmv) to 0.4% (2019 ppmv). Then, a gas sample under equilibrium state, stripped by the equilibrator from fresh tap-water, was measured and is displayed as “Test point 2” in Figure 3a. The calculated gas-phase concentration of “Test point 2” was ~906 ppmv, which was within a reasonable range of CO_2_ concentration dissolved in fresh tap-water. Finally, the reliability of the calibration model was demonstrated. 

### 3.2. System Precision and Accuracy

**Figure 4:**
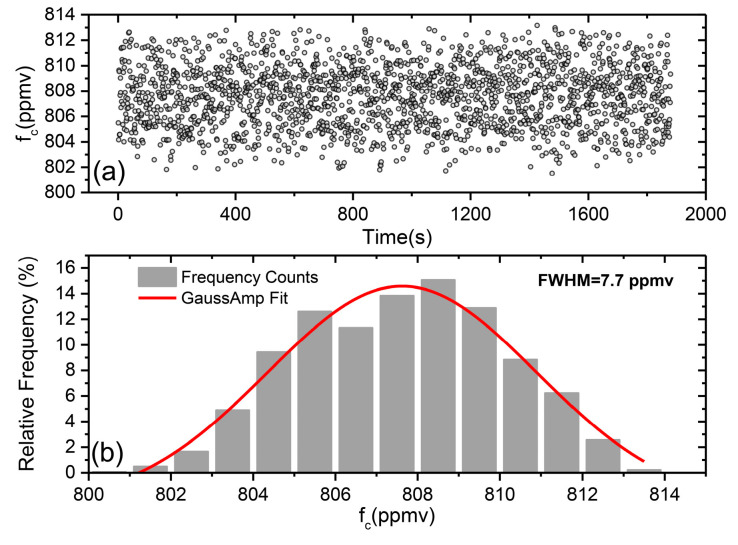
Results of time-series continuous measurement. (**a**) Continuous measurement of a mixture of 810.6 ppmv CO_2_ sealed in the MPGC, a total of 1880 data points acquired with 1 s interval. (**b**) Histogram plot and Gaussian profile, to assess the precision of the system.

To evaluate the precision and the stability of the sensor, a time-series continuous measurement of the gas sample of 810.6 ppmv was carried out over a time period of 31 min. The gas sample was sealed in the MPGC so that its concentration was constant. During the measurement, 1880 data points were obtained with 1 s interval, as shown in Figure 4a. After the comparison of the measured concentration between the beginning and end, the result did not show significant drift. The variation range of the measured concentration was ~802–813 ppmv for the 31 min observation, and the average value of the measured concentration was 807 ppmv. A histogram of the frequency distribution of the measured results is shown in Figure 4b. According to the results fitted by the Gauss profile, the distribution satisfied a Gaussian profile and the full width at half maximum (FWHM) value was 7.7 ppmv, which determined the measurement precision. We took the relative error, half of the ratio of FWHM to the average value of the measured concentration, as the precision of the system, so a precision of 0.5% was obtained.

**Figure 5:**
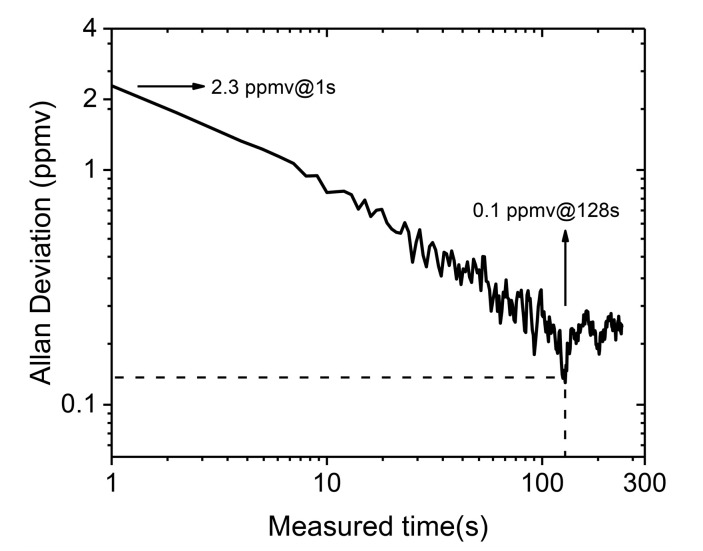
Allan deviation from the time-series measurement.

An Allan analysis was performed to investigate the limit of detection (LOD) and stability, as shown in Figure 5. The Allan variance, along with time, were plotted on a log-log scale. From the plot, we found the sensitivity was 2.3 ppmv with a 1 s integration time, while the LOD was 0.1 ppmv with a 128 s integration time. The results of precision and LOD illustrated that the performance of this system was sufficient to apply it to detection of dissolved CO_2_ in seawater.

The CO_2_ standard gas (810.6 ppmv) was measured discretely with 10 times for the calculation of the system accuracy, as shown in Table 1, and then the accuracy of 0.5% of the system was obtained with the consideration of the uncertainty propagation. In addition, the repeatability of the system was characterized by the experimental standard deviation (σ) obtained under the repeatability condition. So, we acquired the repeatability of the system (σ) of 1.3 ppmv according to the Bessel formula.

### 3.3. System Response Time

**Figure 6:**
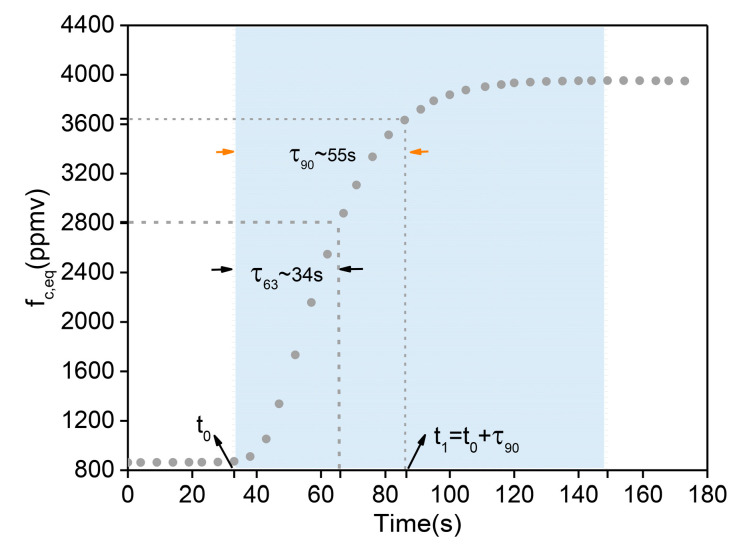
Typical response of the system in conjunction with a home-made sample extraction device whilst the reaction reagent was added to the analyzed water sample at $t_{0}$. $\tau_{90}$ and $\tau_{63}$ were the times taken for the signal to reach 90% and 63% of the final concentration span, respectively. The blue area was the whole rising process from background concentration to maximum concentration.

The dynamic response time is a significant parameter for underway measurements because a faster instrument response implies a higher temporal resolution. This is related to the structure of the MPGC, length of the gas circuit, gas velocity, and efficiency of the sample extraction device. In this experiment, a submerged pump was placed into a container with water open to the atmosphere. Firstly, the container was filled with ~15 L of tap water, and then placed stably in the room for 2 h until the CO_2_ dissolved in the tap water reached a dynamic equilibrium with the atmosphere. Subsequently, a concentration change was performed by way of putting a reaction reagent into the water sample. The reagent was a powdered mixture of the reagents sodium bicarbonate and anhydrous citric acid. The reagent reacted violently with water and a large amount of CO_2_ was released in a short time so that the concentration of dissolved CO_2_ in the water changed rapidly, as shown in Figure 6. The chemical reaction is shown in Equation (6):

(6) $${\mathrm{NaHCO}}_{3}+C_{6}H_{8}O_{7}=C_{6}H_{5}O_{7}{\mathrm{Na}}_{3}+3H_{2}O+3{\mathrm{CO}}_{2}↑$$

As we can obtain from Figure 6, at the beginning of experiment (at 0 s), a background concentration of ~860 ppmv of tap-water in the container was measured by the instrument. At the time $t_{0}$, the reagents were added to the water sample. We observed a distinct rising of the concentration, and the sensor took 55 s and 34 s to reach 90% (at $t_{1}=t_{0}+\tau_{90}$) and 63% of the final concentration span ($\tau_{90}$ = 55 s, $\tau_{63}$=34 s), respectively.

However, in this experiment, $\tau_{90}$ included not only the dynamic response time of the sensor, but also the reaction time between the reagent and water and the mixing time of the upper water and lower water in the container, which were collectively called the delay time [17]. Since these two processes do not exist in natural aquatic systems, the delay time should be subtracted when calculating the response time of the sensor. Finally, we studied 0–90% of the whole rise process, taking 0–10% of the total rise time as the delay time ($\tau_{delay}=$ ~12 s), and 10–90% as the fastest response time of the system ($T_{90}=\tau_{90}-\tau_{delay}$) [17,37]. It was concluded that the fastest response time of dynamic measurement of dissolved CO_2_ in water was ~41 s when the final concentration span was 3079 ppmv.

**Figure 7:**
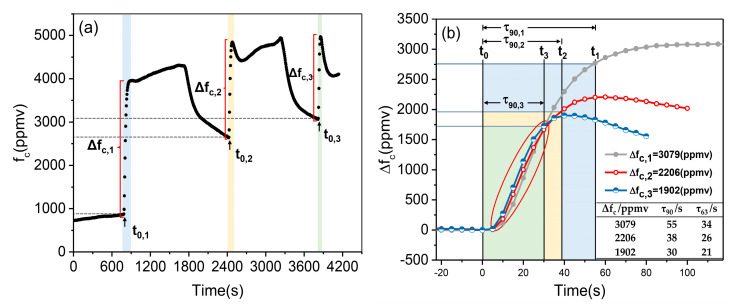
Experimental process and results of response time under different final concentration spans. (**a**) Three concentration rising processes caused by separate doses of reagents. $\Delta f_{c,1}$, $\Delta f_{c,2}$ and $\Delta f_{c,3}$ were the variation range of concentrations after the reagent was put into the water sample. $t_{0,1}$, $t_{0,2}$, and $t_{0,3}$ was the time the reagent was added. (**b**) Comparison of three concentration rising processes ($\Delta f_{c,1}$, $\Delta f_{c,2}$ and $\Delta f_{c,3}$). $t_{1}=t_{0,1}+\tau_{90,1}$, $t_{2}=t_{0,2}+\tau_{90,2}$, and $t_{3}=t_{0,3}+\tau_{90,3}$ were the time taken to reached 90% of $\Delta f_{c}$, respectively.

The concentration increase process described in Figure 6 was only part of the whole experiment, shown in blue area of Figure 7a. In this experiment, three sets of reagents with separate doses were added into the water sample at the times of $t_{0,1}$, $t_{0,2}$, and $t_{0,3}$, respectively. Before the next addition of reagent, the concentration was reduced by replacing part of the water sample in the container with fresh tap water. Therefore, a total of three concentration mutation processes (blue area, yellow area and green area in Figure 7a) were obtained, and their final concentration spans were $\Delta f_{c,1}$ (3079 ppmv), $\Delta f_{c,2}$ (2206 ppmv), and $\Delta f_{c,3}$ (1902 ppmv), respectively. Three rising response processes under the above concentration spans are compared in Figure 7b. Obviously, we can see that there was a tendency between $\Delta f_{c}$ and $\tau_{90}$, which could be described as follows: with the decrease of the value of $\Delta f_{c}$ from 3079 ppmv to 1902 ppmv, the value of $\tau_{90}$ tended to decrease from 55 s to 30 s (as shown in the inset of Figure 7b). Referring to the calculation of $T_{90,1}$ in Figure 6, the $T_{90,2}$ of 27 s and $T_{90,3}$ of 20 s were obtained when $\Delta f_{c,2}$ of 2206 ppmv and $\Delta f_{c,3}$ of 1902 ppmv, respectively. In addition, as shown in the red circle in Figure 7b, the three response processes had good consistency, which indicated that the gas tripped efficiency of the sample extraction device in this system was relatively stable.

In this experiment, the available data of $\Delta f_{c}$ and $\tau_{90}$ was insufficient to support a more quantitative conclusion. However, we were able to confirm that when $\Delta f_{c}$ became very small, the response time of this sensor would become very fast, which was especially important for underway measurements because it meant a higher density of effective data points.

### 3.4. Atmosphere Monitoring in the Laboratory

**Figure 8:**
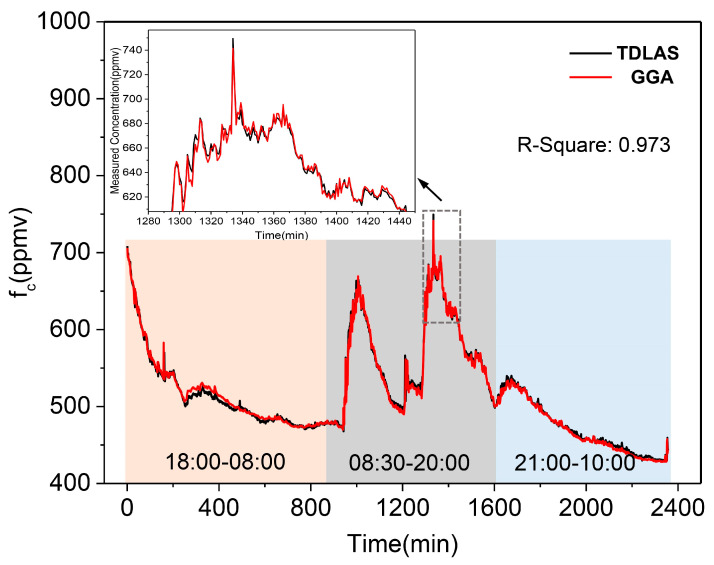
Comparison measurement of ambient CO_2_ in the laboratory between the TDLAS system (black curve) and GGA (red curve). The inset is a detailed plot of a concentration mutation.

The sensor was evaluated by comparison monitoring in a laboratory environment with commercial instruments (GGA, ABB, San Jose, CA, USA), based on off-axis integrated cavity output spectroscopy. For continuous day and night monitoring, the experiment was conducted from 18:00 CST (China) on 5 December 2018, to 10:00 CST on 7 December 2018 (~40 h sampling). We used a Y-joint for sampling for both systems, thus ensuring the consistency of the gas samples. The results were measured by the TDLAS system and by the GGA, and compared in Figure 8. Their linear fitting correlation coefficient value (R-squared) was 0.973.

As can be seen from Figure 8, CO_2_ concentration fluctuations occurred in the laboratory atmosphere, and a high consistency was found for both systems. When the concentration of CO_2_ mutated (as shown in the inset), both the TDLAS system and GGA showed a rapid response to the concentration mutation. The variation range of concentrations was from 428 ppmv to 750 ppmv. During this period, the CO_2_ concentrations were relatively high in the day time (gray area), and then dropped gradually to the minimum value in the early morning (orange and blue areas). There were two increase processes and two decrease processes due to laboratory personnel influence. Although both instruments were able to describe the variation tendency of CO_2_ concentrations accurately, however, the results of the TDLAS system were more detailed, which is particularly significant for underway measurements.

In addition, for the marine real time measurement application, there were some advantages compared with GGA. Firstly, the optical detection part and gas sampling device were integrated in the TDLAS-based system, which realized the underway measurement of dissolved CO_2_ directly. In this way, the integration level and portability of the instrument were improved, and the gas path length between the sampling device and MPGC was saved (gas sample demand can be reduced). With such short gas path length, the “memory effect” of the gas circuit was also weakened. Meanwhile, it weakened the characteristic changes of the gas sample (such as temperature) caused by the long gas path. Secondly, the TDLAS-based system had the better environmental adaptability because of its lower reflectivity requirement of concave mirrors than GGA (more than 99.9%). Therefore, this system was more suitable for long-term gas detection in marine environments, because the gas sample with high salinity and high humidity could decrease the reflectivity of the cavity mirrors.

### 3.5. Field Application

**Figure 9:**
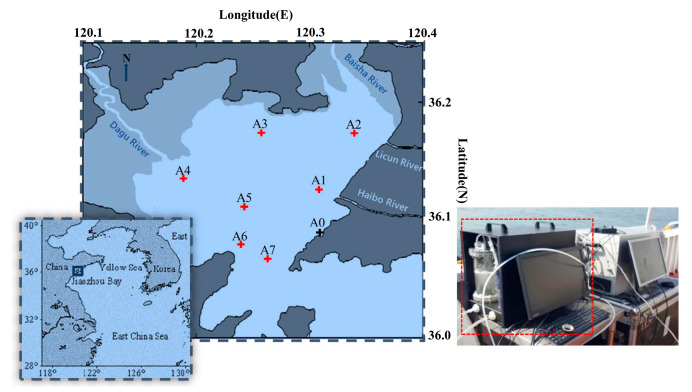
Experimental stations of underway measurement. The A0 station (wharf) was where the employment began. The experiment was performed in an anticlockwise direction. The stations were chosen by the following factors: surface runoff, human activities and special geographic positions (the bay center and the bay mouth).

In order to verify and improve the performance of the system, a field real time application was carried out with the system deployed on deck on 18 September 2019. The measurement site was located in Jiaozhou Bay (35°57′–36°18′ N, 120°04′–120°23′ E), Qingdao, which is a semi-closed bay on the west side of downtown Qingdao, China. As shown in Figure 9, we chose the route according to the following factors: surface runoff (stations A1, A2 and A4), human activities (stations A0 and A3) and special geographic positions (stations A5, A6 and A7), which correspond to estuaries, human living areas, the bay centre and the bay mouth, respectively. 

**Figure 10:**
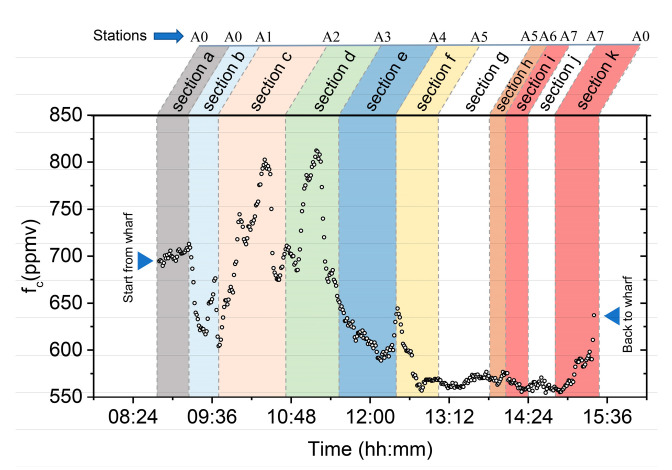
Underway measurement in Jiaozhou Bay. The As mark the measurement stations. For the subsequent analysis, the results were divided into many sections (a-k) with different colors.

The field measurement of the above stations was performed in an anticlockwise direction and the concentration-time plot of this experiment can be found in Figure 10. We found that the $pCO_{2}$ in surface seawater varied greatly (from 550 ppmv to 820 ppmv) in Jiaozhou Bay, and the spatial distribution overall satisfied the following trends: the east and northeast areas of the bay had the highest concentration, and the concentration gradually decreased from the bay center to the bay mouth. Many interesting variations of concentration were measured in the vicinity of these stations, which will be discussed later.

The results were divided into several sections, according to the sequence of stations, for subsequent analysis and discussion. During field monitoring, the instruments were first tested with the surface water within the wharf, allowing for equipment debugging of the system and instrument warm up (section a in Figure 10). In addition, section g and j show the static measurement from the stopped vessel at stations A5 and A7. There were some concentration mutations appeared in Sections b, c, d, f, and the spatial distribution of $pCO_{2}$ could be briefly explained by the following three factors: surface runoff, human activities and geographic position.

- Surface Runoff & Human Activities

On the one hand, surface runoff brings an abundance of diluted water with higher $pCO_{2}$ than seawater in the summer and autumn. On the other hand, rivers carry abundant nutrients that lead to a high primary production of phytoplankton, and cause the $pCO_{2}$ to go down. At the same time, the rivers passing through the human living areas carry an abundance of particulate organic carbon (POC) and biodegradable organic matter (BOM) in the form of sewage, which leads to the enhancement of the aerobic respiration process and causes the $pCO_{2}$ to go up. Actually, in September, primary production of phytoplankton begins to weaken, and aerobic respiration begins to control the distribution of $pCO_{2}$ in the bay. In addition, the seawater near human living areas have higher $pCO_{2}$ levels due to human activity. Therefore, there were some significant increase processes (section b, c, d and f) of the concentration when across the estuaries and human living areas (Stations A1–A4).

- Geographic Position

At the bay center, because it is far away from estuary and human living areas, station A5 has a lower $pCO_{2}$. At the bay mouth (Section h and i in Figure 10), $pCO_{2}$ is at a low level relative to its higher salinity than other areas in the bay. The salinity of seawater at the bay mouth is higher because of its frequent exchange with seawater outside the bay. The tidal variation of Jiaozhou Bay on 18 September 2019, is displayed in Table 2. From ~13:30 p.m. CST, seawater with high salinity begin to pour into the bay, so the salinity of the seawater at the bay mouth increases continuously.

It is worth noting that the distribution of $pCO_{2}$ in the bay was the results of the comprehensive actions of physical and biogeochemical processes. This paper only undertook a brief analysis.

## 4. Conclusions

In this paper, we demonstrated a compact deck-based TDLAS system for dissolved CO_2_ detection in surface seawater, coupled with a home-made headspace equilibrator, allowing continuous and online underway measurements. Both the optical detection part and sample extraction part were fitted together into a portable aluminum alloy chamber that can realize the dissolved CO_2_ measurement directly. The headspace equilibrator used in this work has been improved so that the response time of system was reduced by about 50% compared to traditional “shower head” headspace equilibrator. Under the special design, the two methods of spray and bubble for gas-liquid equilibrium, when the final concentration span was equal to 3079 ppmv, achieved a fast response time on the order of 41 s. For 1902 ppmv, this figure was as little as 20 s. The MPGC was specially designed under the considering the need of small volume and long optical path length. We obtained an empirical equation suitable for the system by deducing theories about the headspace equilibrator, which can convert the concentration of the gas-phase to the liquid-phase. With a time-series (about 31 min) continuous measurement of a gas sample of 810.6 ppmv, a monitoring precision of 0.5% was obtained. The LOD were 2.3 ppmv at 1 s averaging time, and 0.1 ppmv at 128 s averaging time, respectively. Finally, a field underway measurement was carried out and the result was briefly analyzed with the system deployed in Jiaozhou Bay, Qingdao. Our work proved the feasibility of the TDLAS-based system for dissolved CO_2_ rapid detection, and can provide a reference for other researchers engaged in marine sensor development.

Despite thr fact only $pCO_{2}$ dissolved in water is being measured by the sensor in this paper, however, many other dissolved gases could be stripped using the sample extraction device. Therefore, this system also has great potential to detect other dissolved gases, such as methane, and this could be explored in future research.

## Figures and Tables

**Table 1:** Result of discrete measurement of CO_2_ standard gas (810.6 ppmv) ^1^.

**No.**	**1**	**2**	**3**	**4**	**5**	**6**	**7**	**8**	**9**	**10**	**Mean**
**Value**	804.2	806.3	805.9	805.1	807.0	808.9	807.4	806.4	807.0	806.9	806.5

^1^ Units: parts per million by volume (ppmv).

**Table 2:** Tidal chart of Jiaozhou Bay on 18 September 2019 ^1^.

**Tidal Time (hh:mm)**	**08:00**	**09:00**	**10:00**	**11:00**	**12:00**	**13:00**	**13:30**	**14:00**	**15:00**	**16:00**	**17:00**
**Tidal height (cm)**	364	304	257	208	146	98	90	98	150	236	330

^1^ Data from the National Marine Information Center, Global Tidal Service Platform of China. (http://global-tide.nmdis.org.cn accessed on 2 March 2021). Time zone: −800. Tidal datum: 239.0 cm below the mean sea level.

## Data Availability

Data sharing is not applicable to this article.
